# The Role of MicroRNAs in Myeloproliferative Neoplasia

**Published:** 2016-07-01

**Authors:** Shaban Alizadeh, Seyed Ghader Azizi, Masoud Soleimani, Yadollah Farshi, Zahra Kashani Khatib

**Affiliations:** 1 Hematology Department, School of Allied Medicine, Tehran University of Medical Sciences, Tehran, Iran; 2 Hematology Department, School of Medicine, Tarbiat Modares University, Tehran, Iran

**Keywords:** MicroRNA, Myeloproliferative neoplasms, Pathogenesis

## Abstract

MiRs are 17-25 nucleotide non-coding RNAs. These RNAs target approximately 80% of protein coding mRNAs. MiRs control gene expression and altered expression of them affects the development of cancer. MiRs can function as tumor suppressor via down-regulation of proto-oncogenes and may function as oncogenes by suppressing tumor suppressors. Myeloproliferative neoplasias (formerly known as chronic myeloproliferative disorders) form a class of hematologic malignancies demonstrating the expansion of stem cells in one or more hematopoietic cell lines. CML results from an acquired translocation known as BCR-ABL (Philadelphia chromosome). JAK2V617F mutation is present in over 95% of PV, 55% of ET and 65% of PMF cases. Aberrant expression of miR is associated with myeloproliferative neoplasias, pathogenesis, disease progress and response to treatment. MiRs can also be potential therapeutic targets. CML is mainly treated by tyrosine kinase inhibitors such as Imatinib. In addition, altered function of miRs may be used as a prognostic factor in treatment. Resistance to Imatinib is currently a major clinical problem. The role of a number of miRs has been demonstrated in this resistance. Changing expression pattern of miRs can be effective in response to treatment and inhibition of drug resistance. In this paper, we set out to evaluate the effect of miRs in pathogenesis and treatment of MPN.

## Introduction

**Figure 1:**
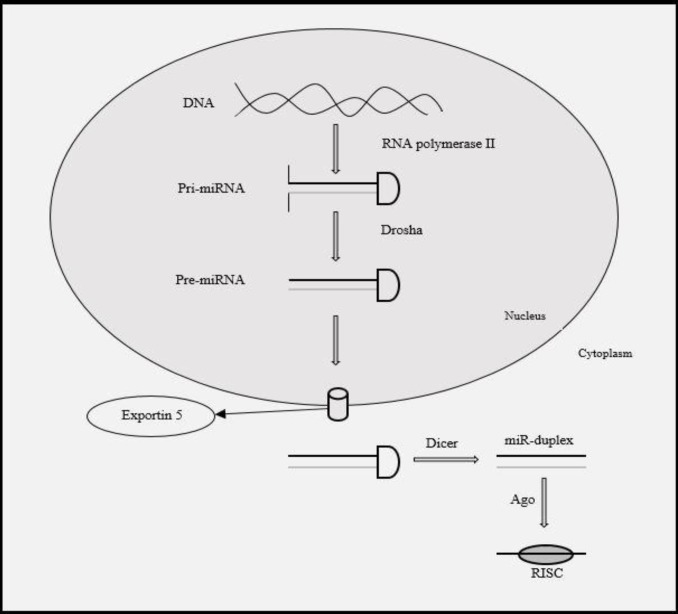
MiRNA biogenesis pathway

 Activation of interfering RNA has been used as a potent and useful biological tool to evaluate the genome function. Several small regulatory RNAs can target this evolutionary conserved pathway to inhibit or suppress the translation of mRNA of interest. These regulatory RNAs include small interfering double stranded synthetic RNAs (siRNA), small hairpin RNAs transcribed by RNA polymerase III or micro RNA (miRs) resulting from RNA polymerase II.^   1 ^ Lin-4 was the first miR detected in Caenorhabditis elegans nematode.^   2 ^ MiRs are non-coding 17-25 nucleotide RNAs.^   3 ^ MiRs do not participate in pathways leading to production of proteins; instead, they regulate the expression of mRNA.^        4 ^ MiRs target approximately 80% of the mRNAs coding for proteins and can be considered as chief regulators of multiple cellular pathways.^        5 ^ MiRs are usually transcribed by RNA polymerase II and the resulting mRNAs are capped and polyadenylated and are known as pri-miRNA.^   6 ^ The first step in production of miR is intranuclear degradation of pri-miRNA, generating a 60-70 nucleotide stem-loop intermediate as miRNA precursor (Pre-miRNA). This is mediated by intranuclear RNase III enzyme of Drosha, which breaks both strands of the stem in sites near the base of the initial stem. Nuclear cut by Drosha gives rise to one end of mature miRNA. The other end is processed in the cytoplasm.^   7 ^ Transfer of pre-miRNA to cytoplasm is mediated by Ran-dependent nuclear transfer receptors of Exportin-5 (Exp5). Pre-miRNA is probably stabilized through interaction with Exp5. ^8^^,^^9^  The pre-miRNA is then degraded by another RNase (Dicer) to generate a 22 bp double-stranded miRNA intermediate. Argonaute (Ago) is a protein which binds the double stranded RNA and participates in the complex formation of single stranded miRNA with Ago, while the other strand is eliminated. The remaining strand depends on the relative thermodynamic stability of both ends of the double stranded intermediate.^   10 ^ This complex (RISC or RNA-induced silencing complex) includes at least one of human Argonaute proteins (Ago1-Ago4).^   11 ^ Argonaute proteins are critical for embryonic development, cell differentiation and maintenance of stem cells.^        12^ Figure 1 shows an overview of miR production steps.

Expression of miRs can be controlled through transcription factors as well as other endogenous and exogenous stimuli. Proteins such as HnRNPA1, SMAD1 and SMAD5 can interact with pre-miRNAs and regulate their processing to mature miRNA. For example, Lin-28 binds let-7 and causes its degradation.^   13 ^ The main function of miRs is mediated through identification of certain binding sites on 3'-UTR in mRNA, inhibiting mRNA translation or causing its destruction.^5^^,^^14^ Each miR regulates more than 100 different mRNAs and over 10,000 mRNAs are directly controlled by miRs.^15^

Although the majority of miRs are intracellular, a significant number of them are observed outside the cells in various body fluids. These miRs are stable and show specific expression profiles in various body fluids. This stability is surprising, since serum and other body fluids are shown to contain ribonuclease enzymes. One of the interesting ideas about the role of miRs is their mediator function in cell-cell communications.^   16 ^ The stability of miRs can be accounted for by discovery of complex lipoproteins containing membrane vesicles of intracellular origin called exosome or micro vesicle. These vesicles contain miRs, mRNAs and protein.^   17 ^ MiRs regulate several biological processes such as apoptosis, insulin secretion, lipid metabolism, stem cell differentiation, cardiac tissue development, muscle differentiation, antigen presentation and aging.^        18 ^

**MicroRNA and cancer**

Cancer models suggest that a small proportion of tumor cells show characteristics of stem cells. These cancer stem cells are responsible for establishing and maintaining the tumor. MiR profile of tumors is reminiscent of stem cells. The expression of several miRs is decreased in tumors but the expression of stem cell miRs is maintained in them.^   19 ^ MiRs control gene expression and altered expression of them can influence the development of cancer. MiRs can function as tumor suppressor via down-regulation of proto-oncogenes and may function as oncogenes through suppression of tumor suppressors.^        12 ^ Experiments show that the majority of miRs are likely to be tumor suppressors. MiR expression patterns in cancer can be used to classify human tumors.^   20 ^

Moreover, miRs affect tumor progression, which involves the metastatic phase of the disease. They participate in several biological processes including control of cell adhesion, migration, invasion and metastasis. MiRs contribute to the regulation of these processes and changing function of miRs can have an impact on metastatic capacity.^   21 ^

On the other hand, when the cell growth is abnormal and apoptosis is impaired, the cells are subject to development of cancer. Several studies suggest that miRs regulate cell growth and apoptosis. For example, miR-15 and miR-16 target mRNA of the anti-apoptotic BCL2 gene, which plays a key role in several human cancers including leukemia, lymphoma and carcinoma. ^22^^-^^24^  The first evidence of miRs involvement in cancer was demonstration of down-regulation or deletion of miR-15a and miR-16-1 in most patients with CLL.^   25 ^ Aberrant regulation of miRs is mediated by several mechanisms including deletion, gene amplification, mutation or regulation of transcription factors that target a specific miR. In addition, the expression of miR can be controlled by two major epigenetic mechanisms, including DNA methylation and histone changes. 2  Approximately, 50% of miR genes are located in loss of heterozygosity, amplification, fragile, viral integration and other genomic sites linked with cancer, indicating the correlation between miRs and cancer.^   26 ^

**The Myeloproliferative neoplasias (MPN)**

Myeloproliferative neoplasias, formerly known as chronic myeloproliferative diseases, form a class of myeloid malignancies showing stem cell related expansion of one or more hematopoietic cell lines. This leads to excessive increase in mature erythrocytes, granulocytes and megakaryocytes. WHO has specified eight MPN types, including chronic myelogenous leukemia (CML), chronic neutrophilic leukemia (CNL), polycythemia Vera (PV), primary myelofibrosis (PMF), essential thrombocytaemia (ET), chronic eosinophilic leukemia (CEoL), mastocytosis and unclassified myeloproliferative neoplasias.^27^^,^^28^

CML is developed due to an acquired translocation of BCR-ABL or Philadelphia chromosome, a hybrid oncogene with permanent tyrosine kinase activity. BCR-ABL negative MPNs are conditions associated with acquired tyrosine kinase mutations. JAK2V617F mutation is present in more than 95% of PV, 55% of ET and 65% of PMF cases. MPN patients have hepatomegaly and splenomegaly along with the proliferation of a cell line or a combination of cytopenia with increase in peripheral blood cell count. The majority of patients are in the fifth to seventh decades of their lives.^   28 ^ Primary steps of MPN may not be associated with hematological symptoms.^   29 ^

**CML**

Chronic myeloid leukemia (CML) is one of the most common leukemias in the world and over 90% of its cases are associated with the presence of Philadelphia chromosome.^   30 ^ CML is a clonal myeloproliferative disease related to HSCs, clinically recognized by triple-phase clinical presentation as well as translocation between chromosome 9 and 22 (Philadelphia chromosome). Joining BCR gene from chromosome 22 with ABL gene on chromosome 9 causes dysregulation of internal non-receptor tyrosine kinase activity of ABL gene. The hybrid oncogene of BCR-ABL is the main cause of CML development by controlling processes like cell proliferation, adhesion and survival.^   31 ^ The chronic primary phase of the disease is gradually changed to invasive phase followed by blastic phase or blast crisis. ABL-BCR is an active etyrosine kinase targeted by tyrosine kinase inhibitors such as Imatinib, Nilotinib and Dasatinib.^   32 ^

**Figure 2:**
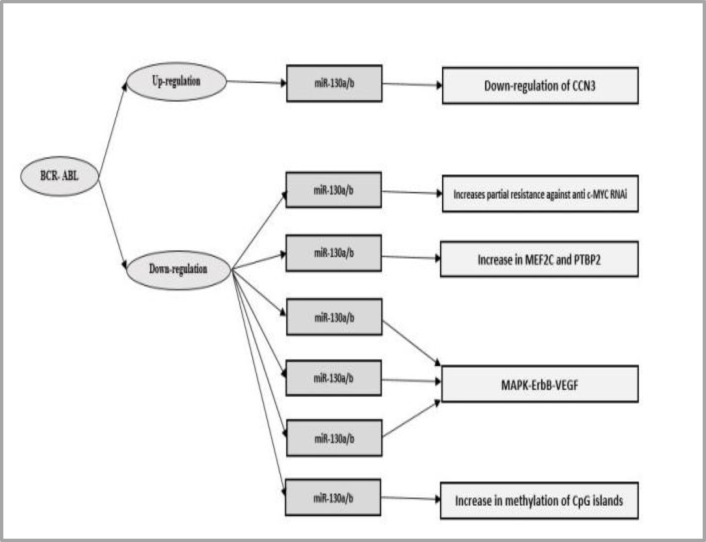
BCR-ABL associated miRNAs

Abnormal expression of miR is associated with CML and is involved in pathogenesis, disease progression and response to treatment. It may also be a potential therapeutic target for treatment.^   33 ^ In Table 1, a list of miRs undergoing altered expression in CML has been presented and Figure 2 presented BCR-ABL associated miRs.

C-myc expression is increased in K562R cell line resistant to Imatinib, which in turn reduces the expression of miR-144/451.^   30 ^ On the other hand, bilateral regulatory loop between BCR-ABL and miR-451 maintains the leukemic state of CML cells.^   34 ^

CCN3 is generally considered as a negative regulator of growth and functions as a tumor suppressor gene in solid tumors. In addition, it plays a key regulatory role in biological processes. CCN3 is subject to negative regulation in CML, which returns to normal after treatment with Imatinib. Negative regulation of CCN3 in CML is mediated by BCR-ABL-dependent miRs. MiRs-130a, 130b, 148a, 212 and 425-5p are significantly decreased in K562 cells in which BCR-ABL has been knocked down. Decrease in BCR-ABL is accompanied by an impressive increase in mRNA of CCN3 and the negative regulation of CCN3 observed in CML may be mediated by the expression of miR-130a/b.^35^^,^^36^

Ral-A is a downstream molecule of BCR-ABL in Ras signaling pathway and is a direct target for miR-181a. Ral-A is a member of small G protein family involved in development of tumor, invasion and metastasis. High expression level of miR-181a effectively inhibits the cell growth inducing growth arrest in G2 phase and apoptosis via targeting Ral-A in K562 cells. The expression of miR-181a in hematopoietic stem and progenitor cells regulates the development of blood cells.^   37 ^ BCR-ABL mediated suppression of miR-223 leads to activation of MEF2C and PTBP2 in CML. The expression of this miR causes increased myeloid differentiation. The expression of miR-223 is controlled by three transcription factors of C/EBP α, PU-1 and NFI-A.^   38 ^

Low expression level of miR-196b amplifies the expression of BCR-ABL and HOXA9 oncogenes in CML. CpG islands show a higher level of methylation in CML patients compared with healthy subjects indicating that low expression of miR-196b may be associated with increased methylation in CpG Islands. BCR-ABL1 and HOXA9 are target genes of miR-196a. MiR-196b causes reduced level of BCR-ABL1 and HOXA9 proteins, decreased cell proliferation and delayed cell cycle. Low expression level of miR-196b can cause up-regulation of BCR-ABL1 and HOXA9, leading to progression of CML.^   34 ^

MiRs can be encoded in transcript clusters as polycistronic pri-miRNA clusters. MiRs-17-3p, 18a, 19a, 20a, 19b and 92-1 are transcribed from an intron in C13-25 locus at 13q31-32. In BCR-ABL positive cell lines, the expression of miRs encoded in polycistronic miR-17-92 cluster is subject to down-regulation. Increased expression of polycistronic miRs in K562 cells due to Lentivirus leads to increased proliferation, relative resistance to anti-c-MYCRNAi and increased sensitivity to apoptosis caused by Imatinib. MiRs-17-92 undergo increased expression in primary CD34^+^ CML cells in the chronic phase but not during blast crisis, raising a potential collaboration between BCR-ABL c-MYC-miR-17-92.^   39 ^

MiR-30a suppresses the proliferation of leukemic cells in vitro through the interruption of cell cycle and functions as a tumor suppressor against CML by expression regulation of BCR-ABL1. Increased expression of miR-30a in K562 cells reduces ABL1 and BCL-ABL1 protein levels, causing significant reduction in cell proliferation rate and cell cycle arrest between G1 and S phases.

Down-regulation of miR-30a in cells with overexpression of miR-30a restores ABL1 and BCR-ABL1 protein levels, accelerating cell entry into S phase.^   34 ^

Increased expression of miR-203 in BaF3 positive BCR/ABL cells with T315I mutation inhibits cell growth and colony forming ability and miR-203 increases sensitivity to Imatinib in BaF3-BCR/ABL cells. MiR-203 is significantly down-regulated in CML patients and the imposed expression of miR-203 is able to inhibit the proliferation of BCR/ABL cells positive for T315I mutation.^   40 ^ In CML cells, miR-31, miR-155 and miR-564 are down-regulated and down-regulation of them is dependent upon the activity of BCR-ABL. CML is considered as the main disease related with these miRs. MAPK, ErbB, mTOR and VEGF are the major molecular pathways associated with this expression pattern.^   41 ^

MiR-29 family includes three members: miR-29a, miR-29b and miR-29c. Several oncogenes are silenced by miR-29 including anti-apoptotic MCl-1, upstream inhibitors of p53 like p58α and CDC42, DNA methyltransferases and extracellular matrix proteins. This miR, which is down-regulated in CML blast crisis is associated with apoptosis, cell cycle and cell proliferation indicating miR-29b as a critical regulatory molecule in the initiation and progression of cancer.^   42 ^ Regulation of human RNase-L via miR-29 may indicate an oncogenic role in CML. RNase-L is involved in antimicrobial and tumor suppressor activities. RNase-L is post translationally expressed by 3'UTR. MiR-29 family members suppress the expression of RNase-L protein. MiR-29 family members function via four target regions in 3'UTR RNase-L. Loss of RNase-L function in K562 cells causes proliferation suppression in vitro.^   43 ^

MiR-30a is a potent inhibitor of autophagy through expression down-regulation of Beclin1 and ATG5. Knocking down this miR through 30a antagomir increases the expression of Beclin1 and ATG5, inhibiting the cytotoxicity exerted by Imatinib.^   44 ^

ABCG2 (ATP-binding cassette transporter) plays a key role in cancer. This protein appears to play an important role in transport of various drugs.^   45 ^ ABCG2 is increased in K562 cells after prolonged treatment with Imatinib. MiRs 212 and 328 are inversely related to ABCG2 expression. MiR-212 targets the 3'-UTR region in ABCG2. AntimiR-212 causes up-regulation of ABCG2 protein while the effect of anti-miR-328 is impotent in this regard.^   46 ^

Hedgehog (Hh) signaling pathway is a functional pathway for cancer stem cells. It is an evolutionary conserved pathway that plays an important role in early hematopoiesis in adults. Secretory Hh proteins bind to Ptch receptor, which passes through the membrane seven times. Ptch exerts a suppressor function on Smo. Smo (Smoothened receptor) is a receptor passing seven times through the membrane. When Hh proteins bind Ptch receptor, the inhibitory effect of Smo is relaxed. The intracellular signaling pathways ultimately lead to activation of Gli transcription factors and augment the expression of target genes such as Gli1, Ptch1, cyclin D1 and Bcl-2. Signaling caused by Gli leads to cell cycle stimulation, apoptosis inhibition, preservation of self-renewal of stem cells and differentiation regulation of tissue stem cells. Overexpression of miR-326 leads to down-regulation of Smo, resulting in reduced cell proliferation as well as increased apoptosis in CD34^+^ CML cells. Loss of this pathway disrupts the development of CML cells due to BCR-ABL, reducing the number of CML stem cells. ^47^^,^^48^ 

Down-regulation of has-miR-10a in CD34^+^ CML cells increases USF2-dependent cell growth. Has-miR-10a, has-miR-150 and has-miR-151 are down-regulated in CML cells but miR-96 is up-regulated. Down-regulation of has-miR-10a is not dependent upon the activity of BCR-ABL1 and plays a role in increased cell growth in CML. USF2 (upstream stimulatory factor 2) is a potential target of has-miR-10a and increased expression of USF2 increases cell growth and is associated with reduced expression of has-miR-10a. Down-regulation of has-miR-10a may increase USF2 and may be involved in increased cell proliferation in CML.^   49 ^

Inhibition of miR-21 by miR-21 related antisense in K562 cells suppresses cell migration, increases cell apoptosis, suppresses cell growth and up-regulates the expression of PDCD4 tumor suppressor gene. Pre-miR-21 increases cell migration and increases cell apoptosis without affecting cell proliferation. PDCD4 is a functional target for miR-21 in K562 cells. MiR-21 may have an oncogenic role in cellular process of CML and antisense inhibition of miR-21 may be beneficial as a treatment for CML.^   50 ^

**BCR-ABL negative Myeloproliferative neoplasias**

JAK2V617F mutation detection in the majority of patients with negative Philadelphia chromosome was an important progress in understanding MPN pathobiology. JAK2 pathway regulates normal myelopoiesis via signaling of class 1 cytokine receptors such as erythropoietin receptor, thrombopoietin receptor and G-CSF receptor.^   60 ^

The effect of JAK2V617F mutation depends on the cellular context. In non-hematologic 293T cells, JAK2 V617F protein is highly phosphorylated in the absence of exogenous stimuli. In hematopoietic cell lines, such as BaF3 or Ut7, the impact of mutations is very accurate and includes reduced apoptosis tendency and moderate proliferation.^   61 ^ Mutation in JAK2 is seen in nearly all patients with PV and in a high proportion of patients with ET and PMF.^   62 ^ Jak2V617F mutant cells show automatic activation of multiple cell signaling pathways, especially JAK/STAT as well as cytokine independent proliferation and maturation.^   63 ^ In addition to JAK2 mutation, mutation in MPL, LNK, CBL, Tet2, Asxl1, Idh, Ikzf1, Ezh2, Dnmt3A, TP53 or SF3B1 may also be observed in these patients.^   64 ^ PV, ET and PMF represent the Philadelphia negative classic myeloproliferative neoplasias. ET and PV are highly sensitive to cytokines such as erythropoietin (Epo), thrombopoietin (Tpo), Interleukin-3 (IL-3) and SCF, enabling proliferation and differentiation of erythroid progenitor cells in the absence of Epo (the so-called endogenous erythroid colonies or EEC).^   65 ^

In ET, mutated and wild-type clones are simultaneously present without a change in the ratio of mutant to wild-type cells during long-term monitoring. In contrast, steady increase is seen in population of stem or progenitor cells bearing mutation in JAK2 along with reduced number of normal stem cells.^   66 ^

Hypermetylation of miR-203 is not specific for Philadelphia-positive leukemias but is present in Philadelphia negative MPN.^   67 ^ MiR-28 targets the 3' UTR region of thrombopoietin receptor (MPL) and suppresses its translation. The expression of miR-28 in CD34^-^ megakaryocytes inhibits terminal differentiation. The expression of miR-28 is increased in platelet fraction of MPN patients.^   68 ^

MiR-433 is ectopically expressed in MPNs, suppressing the growth and differentiation of hematopoietic cells.Gbp2, the new target for miR-433, is subject to down-regulation during normal erythropoiesis, regulating the proliferation and differentiation of erythroid cells in TF-1 cells. Expression of miRs-134, 214 and 433 is not affected by changes in the activity of JAK2, suggesting the involvement of different signaling pathways in aberrant regulation of these miRs in MPN.^   69 ^ Abundance of morphologically abnormal megakaryocyte is a feature of Ph^-^ MPNs. MiRs 17-5p, 20a and 126 are expressed in Ph^-^ MPNs, while the low or absent expression of miR-10a is associated with megakaryocytic expression of HOXA1 protein.^   70 ^ MiR-433 is ectopically expressed in MPNs and suppresses the growth and differentiation of hematopoietic cells.^   71 ^

**Polycythemia Vera**

Polycythemia Vera is a clonal disease that has similarities with the proliferation of clonal myeloid cells. Clinical and pathological features of PV are close to ET and PMF. In the most common case, PV is characterized by erythrosis, which is variably associated with leukocytosis and thrombocytosis. PV is developed due to mutation in a single hematopoietic precursor. This mutation affects multipotential stem cells and leads to abnormal growth of precursor cells, enabling them to form erythroid colonies in the absence of exogenous erythropoietin.^   71 ^ MiR-451 is highly expressed in erythrocytes and reticulocytes but not in platelets, granulocytes, monocytes and lymphocytes. MiR-144 and miR-451 are regulated by GATA1, the specific transcription factor of erythroid series. Down-regulation of a number of erythropoiesis related miRs like 15 a, 150, 221, 223, 24 and 103 results in increased erythropoiesis stimulants, while the up-regulation of miRs-320, 451 and 144 inhibits the down-regulators of erythropoiesis.^   72 ^

The level of miR-451 and miR-150 is disrupted in differentiated erythroid PV cells. Up-regulation of miR-451 and down-regulation of miR-150 is associated with development of erythroid maturation in K562 cells. Imposed miR-451 expression increases erythroid differentiation. Inhibition of miR-150 reduces hemoglobin production in K562 cells. Target genes regulated by miR-451 and miR-150 include Ube2H, ARPP-19 as well as MS4A3, AGA and PTPRR, respectively.^   73 ^ Up-regulation of miR-451 and down-regulation of miR-150 has a positive effect on the expression of GATA-1, FOG-1, EKLF, CD71 and CD235a and effectively induces the production of hemoglobin.^   74 ^

**Figure 3:**
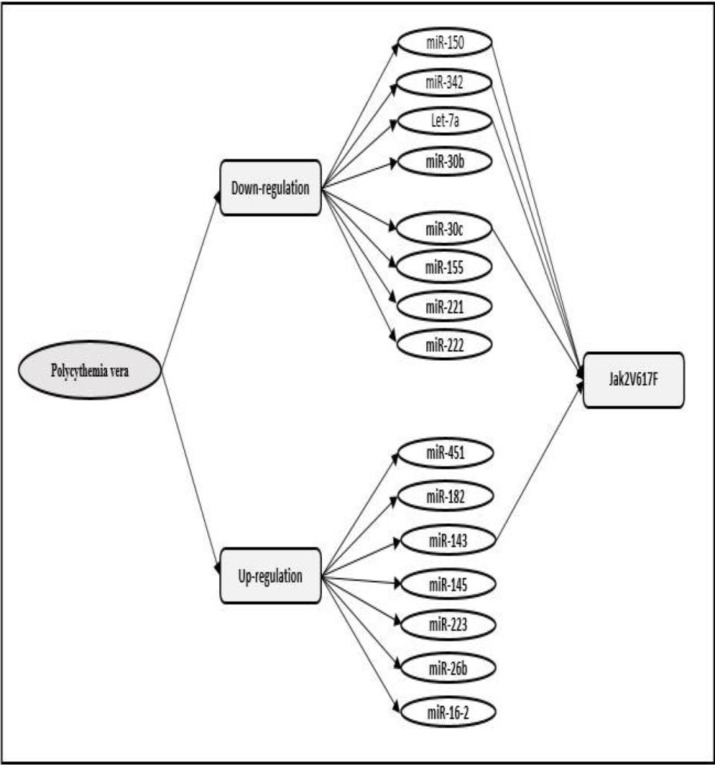
MiRs effective in PV

In PV, down-regulation of let-7a and up-regulation of miR-182 in granulocytes, up-regulation of miRs-143, 145 and 223 in mononuclear cells, up-regulation of miR-26b in platelets and down-regulation of 30b, 30c and 150 are observed in reticulocytes. JAK2V617 is often positively related with the expression of miR-143 and is negatively correlated with let-7a, miR-30c, miR-342 and miR-150. ^75^^,^^76^  Overexpression of miR-16-2 is involved in abnormal hematopoiesis in PV. Imposed expression of miR-16 in normal CD34^+^ cells stimulates the proliferation and maturation of erythroid cells. CD34^+^ cell contacts with interfering RNA against pre-miR-16-2 reduces the number of erythroid colonies and inhibits the formation of erythropoietin independent colonies. Overexpression of miR-16 is not associated with the activation of JAK/STAT pathway.^   77 ^ Down-regulation of miRs-150, 155, 221 and 222 and up-regulation of miRs-451 and 16 is observed in end stages of erythropoiesis and biphasic regulation of miR-339 and miR-378 is seen in normal hematopoiesis.^   78^ Figure 3 shows miRs which are effective in PV.

**Table 1:** MiRNAs changed in CML

**Mir**	**Expression**	**Function**
Has-miR-10a	Reduced	Down-regulation of has-miR-10a, independent of BCR-ABL1 activity, participation in cell growth in CML^ 49 ^ USF2 a potential target of has-miR-10a and increases cell growth^ 49 ^
MiR-17-92 cluster	Reduced	Increased expression of polycistronic miR in K562 cells, an increase in proliferation, relative resistance against RNAi, anti c-MYC, increasing susceptibility to cell death due to Imatinib^ 39 ^
MiR-21	Increased	Considerably reduced in K562 cells subject to BCR-ABL knockdown^ 34 ^
MiR-26b	----	Can participate in increased expression of gamma-globin chain gene in K562 cells^ 51 ^
MiR-29b	Reduced	Increased expression of it decreases the expression of BCR/ABL in mRNA and protein levels^ 43 ^
MiR30a	Reduced	Increased expression of it as a tumor suppressor against CML in K562 cells reduces protein levels of ABL1 and BCR-ABL1^ 33 ^
MiR-31 and 564	Reduced	Down-regulation of this miR is dependent upon the activity of BCR-ABL^ 41 ^
Has-miR-96	Increased	Increased in CML cells^49^
MiR-130a/b	Increased	Decreased in K562 cells treated with Imatinib Decreased BCR-ABL is associated with significant increase in CCN3 mRNA^ 34 ^
MiR-138	Reduced	Overexpression of it leads to down-regulation of BCR-ABL^ 52 ^
MiR-148a	Increased	Significantly decreased in BCR-ABL knocked out K562 cells^ 34 ^
MiR-155	Reduced	Down-regulation of miR-155 by a microRNA inhibitor as miR suppressor can increase the expression of alpha globulin chain in K562 cell line^ 53 ^ Down-regulation of miR-155 by LNA inhibitor can reduce cell growth and proliferation in PC12 cell line^ 54 ^
MiR-181a	Reduced	Down-regulates RalA oncogene and participates in growth inhibition and apoptosis in CML^ 36 ^
MiR-196b	Reduced	Low expression of miR-196b stimulates the expression of BCR-ABL and HOXA9 oncogenes in CML^ 38 ^
MiR-203	Silent	Restoration of it reduces ABL1 and BCR-ABL1 expression as well as cell proliferation^33^^,^^55^ Increases sensitivity to Imatinib in BaF3-BCR/ABL cells^ 40 ^ Imatinib causes demethylation of promoter region of miR-203, which leads to low BCR-ABL gene expression and loss of proliferation capacity of leukemic cells^ 56 ^ Hypermethylated gene in CML^ 57 ^
MiR-212	Increase	Causes up-regulation of ABCG2 protein expression^ 46 ^
MiR-217	Reduced	Imposed expression of miR-217 inhibits the expression of DNMT3A^ 58 ^
MiR-223	Reduced	Suppression of miR-223 by BCR-ABL leads to activation of MEF2C and PTBP2 in CML^ 37 ^
MiR-326	Reduced	Increased expression of miR-326, inhibits cell proliferation and can induce apoptosis in CD34^+^ CML cells^ 47 ^
MiR-425	Increased	Dramatically reduced in K562 cells knocked out for BCR-ABL^ 34 ^

**Primary myelofibrosis**

In PMF, clonal myeloproliferation is associated with reactive bone marrow fibrosis, osteosclerosis, angiogenesis, extramedullary hematopoiesis and abnormal expression of cytokines. Clinical features of PMF include severe anemia, marked hepatosplenomegaly, cachexia, bone pain, thrombosis and hemorrhage.^   64 ^ Mutation in JAK2 gene is observed in 35 to 50% of patients.^   79 ^ The level of miRs-31, 150 and 95 is significantly lower in PMF, while miR-190 is significantly higher than the control samples, PV or ET.

MiRs-34a, 342, 326, 105, 149 and 147 is similarly reduced in PMF and PV in comparison with control.^   80 ^ MiR-4319 is down-regulated during conversion of PMF to AML and its target gene (SETBP1) is increased.^   81 ^ High megakaryocytic expression levels for miR-223 is observed in PMF, and increased miR-146b can be seen in cellular stage of PMF.^   82 ^ Furthermore, ectopic expression of miR-10a is seen in PMF and PV.^   83 ^

**Essential thrombocytaemia**

ET is a chronic myeloproliferative neoplasia characterized by persistent thrombocytosis, megakaryocytic BM hyperplasia, increased risk of thrombosis and hemorrhage.^   84 ^ There is a high risk for ET conversion to myelofibrosis or acute myeloid leukemia but thromboembolic problems with an incidence of 11-25% are the most important complications that can have a major impact on patients’ survival.^   85 ^ Mutation in JAK2V617F is observed in 50% of patients.^   68 ^

MiRs-34a, 342, 326, 105, 149 and 147 are decreased in ET compared with control.^   80 ^ High expression of megakaryocytic levels of miR-223 is observed in PMF and ET.^   82 ^ Ectopic expression of MiRs-10a and 150 is seen in ET, PMF and PV.^   83 ^ MiRs-133a and 1 are down-regulated in neutrophils from patients with PV and ET. These two miRs are located on the same bicystronic unit on chromosome 18 and are simultaneously transcribed. Down-regulation of these miRs is involved in proliferation of hematopoietic cells.^   86 ^

**MiRNAs related to treatment**

Treatment of CML is based on tyrosine kinase inhibitors, especially Imatinib.^   87 ^ Imatinib has revolutionized CML therapy by suppressing the tyrosine kinase activity of ABL-BCR, which leads to tumor cell death.^   88 ^

**Mechanism of treatment by miRNAs**

Several miRs are down-regulated in CML including miRs-150, 146a, 143-3p, 199b-5p and 138. These miRs rapidly return to normal levels in patients treated with Imatinib. ^52^^,^^89^  The expression of miR-130a and miR-130b is reduced in K562 cells treated with Imatinib but the expression of CCN3 is increased in them. CCN3 is typically a negative regulator of growth.^   35 ^

Firatligil et al. evaluated the expression levels of miR-17 as an oncogene in Imatinib sensitive and resistant CML cells. The results show significantly increased expression of miR-17 in Imatinib sensitive and resistant CML cells compared to peripheral blood mononuclear cells. On the other hand, a dramatic reduction was observed in the levels of miR-17 in response to Imatinib, Nilotinib and Dasatinib.^        90 ^

Ferreira et al. showed that Dasatinib regulates the expression of miR-let-7e, miR-let-7d, miR-15a, miR-16, miR-21, miR-130a and miR-143-3p, while Imatinib regulates the expression of miRs-15a and 130a and the expression of miRs16, 130A and 145 is regulated by Nilotinib. They also observed high levels of miRs-15a, 130b and 145 and decreased expression of miRs-16, 26a and 146a in chronic phase compared with controls. Patients in the acute phase showed a low expression level of miRs let-7d, 15a, 16, 29c, 142-3p, 145 and 146a in comparison with the chronic phase.^   91 ^

Imatinib results in demethylation of the promoter region of miR-203, which leads to lower gene expression of BCR-ABL and loss of expansion capacity in leuckemic cells.^   57 ^ In addition, miR-203 increases sensitivity to Imatinib in BaF3-BCR/ABL cells.^   40 ^ Flamant et al. showed that after two weeks of treatment with Imatinib, the expression of miRs 150 and 146A increased and that of miR-142-3p and 199b-5p decreased.^   92 ^

Xu et al.^52^ reported a network of BCR-ABL/GATA1/miR-138 in CML. MiR-138 is subject to down-regulation in K562 and primary CML cells and returns to normal after treatment with Imatinib. Tumor suppressor activity of miR-138 is accounted for by the induction of arrest in G0/G1 phase, suppression of cell proliferation, colony formation of CFU-GM and increased induction of apoptosis in K562 and Ku812 cells.

Increased expression of miR-138 leads to BCR-ABL down-regulation. They showed that ABL and BCR-ABL are the genes regulated by miR-138. Instead of binding to 3'-UTR region in mRNA related to AML, miR-138 binds the coding region. In addition to ABL, CCND3 is another target of miR-138, binding to 3'UTR and neutralizing its mRNA. They also demonstrated that up-regulation of miR-138 through treatment with Imatinib is associated with increased activity of GATA1, which binds the promoter of miR-138. As a result, miR-138 is a tumor suppressor miR with a low level of expression in CML. MiR-138 expression is activated by GATA1 and inhibited by BCR-ABL. Thus, miR-138 is a tumor suppressor through the BCR-ABL/GATA1/miR-138 network, indicating CML pathogenesis and its clinical response to Imatinib.^   52 ^

Anti miR-21 oligonucleotide (AMO-miR-21) and arsenic trioxide (ATO) cause inhibition of growth, apoptosis and G1 phase arrest in K562 cells. AMO-miR-21 significantly enhances growth inhibition by ATO and apoptosis but has no effect on G1 phase. AMO-miR-21 sensitizes K562 leukemic cells to ATO through the induction of apoptosis, partly due to positive regulation of PDVD4 protein level. When AMO-miR-21 and ATO are simultaneously administered, therapeutic dose of ATO is reduced. This can lead to low nonspecific effects and high tolerance as well as likely reversed drug resistance in leukemia.^   93 ^ Long-term exposure of K562 cells to Dasatinib together with 5-Aza-2'-deoxycytidine inhibits the proliferation potential of these cells in association with increased expression of miR-217 and down-regulation of DNMT3A in vitro.^   59 ^

MiR-153 sensitizes K562 cells to apoptosis induced by As2O3. Furthermore, miR-153 is subject to down-regulation in K562 cells resistant to treatment with As2O3. When the cells are treated with As2O3, significant apoptosis is seen in cases with high miR-153 levels.^   94 ^

**Figure 4:**
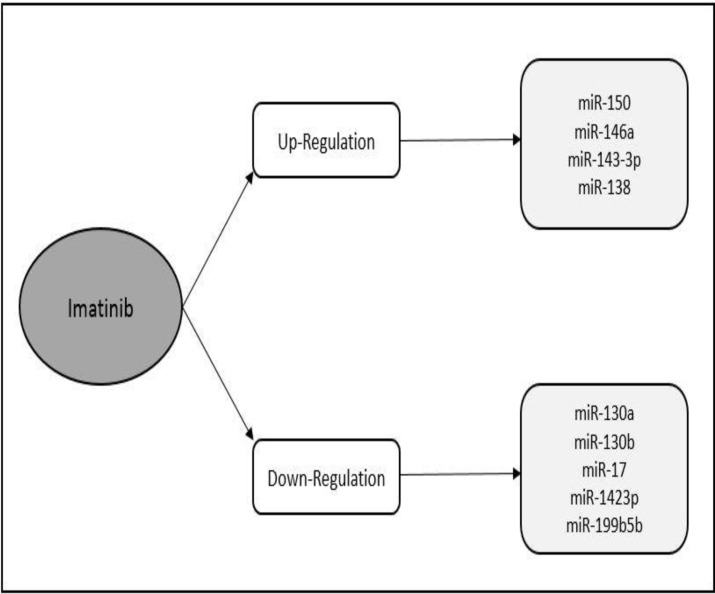
The effect of Imatinib on various miRNAs

Homoharringtonine (HHT) is a traditional Chinese medicine successfully used to treat leukemia. It inhibits the production of G1 and G2 proteins, inhibits cell differentiation and stimulates apoptosis. Laboratory studies have indicated a cooperative activity between HHT, Ara-C and IFN-α. Fox-M1 is the major regulator of cell proliferation and apoptosis. MiR-370 significantly intensifies HHT mediated cell apoptosis and miR-370 and HHT synergize in effective expression of FoxM1. MiR-370 controls a number of genes including the Wilm’s tumor gene on chromosome X, Insulin receptor substrate 1, Forkhead box protein O1 and FoxM1.^   95^ Figure 4 illustrates the effect of Imatinib on various miRs.

**Drug resistance**

Resistance to Imatinib has turned out to be a major clinical problem.^   88 ^ Resistance to Imatinib may be caused by increased activity of BCR-ABL due to increased expression of BCR-ABL gene or point mutation of it as well as activation of other survival related pathways such as the activation of lyn, ERK, PI3K, MDR1 and COX2 induction.^   30 ^ In addition, miRs with modified function may be used as prognostic factors in therapy.^   87 ^

C-myc expression is increased in Imatinib resistant K562R cells. Reintegration of miR-144/451 or removal of Myc can sensitize Imatinib resistant cells to apoptosis.^   30 ^ MiR-30a intensifies resistance to Imatinib in CML, suppressing autophagy through down-regulation of Beclin1 and Atg5 expression. ^34^^,^^44^  MiR-181C targets such gene as Pbx3, Hsp90B1, Nmt2 and RAD21 that are associated with drug resistance, on the other hand, miR-181C is down-regulated in Imatinib resistant CML.^   96 ^

Long-term exposure of K562 cells to inhibitors of tyrosine kinase effect of BCR-ABL causes drug resistance in conjunction with an increase in the level of DNA Methyl transferase (DNMT) and reduction in the level of miR-217. Imposed expression of miR-217 inhibits the expression of DNMT3A by a binding site in miR-217 to 3' UTR region related to DNMT3A and by sensitizing the cells to growth inhibition by TKI.^   59 ^ Expression of miRs-26a, 29c, 130b and 146a in Imatinib resistant cells is down-regulated compared to patients responding to Imatinib therapy.^        90 ^

**Figure 5:**
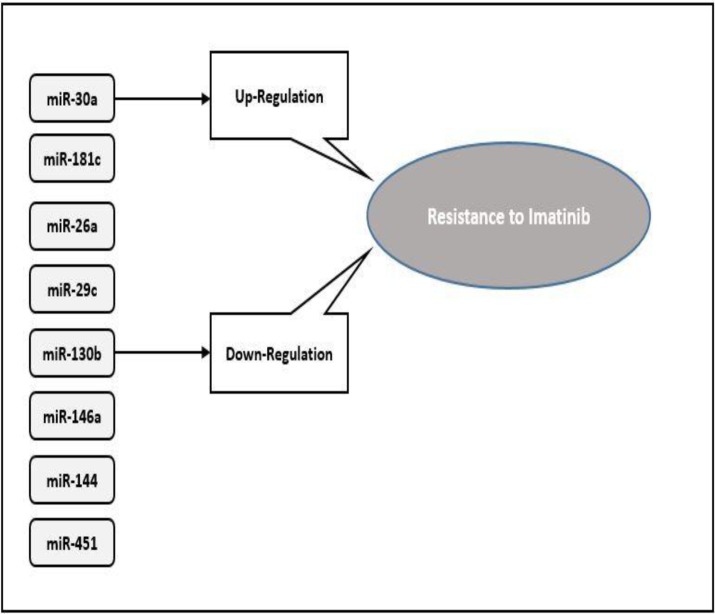
MiRNAs associated with resistance to Imatinib

MiR-181c targets genes such as PBX3, HSP90B1, NMT2 and RAD21, which are associated with drug resistance.^   96^ Figure 5 shows miRs associated with resistance to Imatinib.

## CONCLUSION

 Evaluation of the function of miRs has dramatically changed our understanding of cancer mechanisms. MiRs can be used for diagnosis, stage classification, prognosis and treatment response or resistance in various cancers including MPNs. Targeting specific miRs can be taken advantage as a specific treatment by affecting the expression of genes of interest. At present, the mechanism of action of many miRs in several cancers is unknown, demanding further studies in this field. 

MiR based treatments are currently in clinical trial stage, which promise more specific treatments in the future. Despite recent advances in the study of structure and function of miRs, the role of these molecules in MPN has many dark points and our knowledge in this field is very limited. These findings reflect the fact that each MPN type has its specific expression pattern of miRs, which is involved in its pathogenicity. Further studies in this context can increase our understanding of MPNs and provide us with newer and more specific treatment methods**.**
